# Supplementary material for: Apolipoprotein B is associated with CT-angiographic progression beyond low-density lipoprotein cholesterol and non-high-density lipoprotein cholesterol in patients with coronary artery disease
Source: Lipids Health Dis. 2023 Aug 9;22:125. doi: 10.1186/s12944-023-01872-6 (PMC10410799; doi:10.1186/s12944-023-01872-6)
Supplement: Supplementary file 1 — Supplementary Material 1 [file 12944_2023_1872_MOESM1_ESM.docx]

Supplementary Table 1. Baseline characteristics of patients categorized by concordant and discordant values of apoB versus non-HDL-C

| Variables | Low apoB/  Low Non-HDL-C | Low apoB/  High Non-HDL-C | High apoB/  Low Non-HDL-C | High apoB/  High Non-HDL-C | *P* value |
| --- | --- | --- | --- | --- | --- |
| N | 197 | 66 | 75 | 206 |  |
| Age, years | 67.4 ± 9.9 | 64.9 ± 9.9 | 63.0 ± 10.7 | 62.0 ± 11.0 | < 0.001 |
| Male, n (%) | 119 (60.4) | 27 (40.9) | 49 (65.3) | 112 (54.4) | 0.015 |
| BMI, kg/m^2^ | 24.1 ± 2.9 | 24.6 ± 3.0 | 25.0 ± 2.9 | 25.1 ± 2.8 | 0.009 |
| Hypertension, n (%) | 150 (76.1) | 46 (69.7) | 49 (65.3) | 150 (72.8) | 0.318 |
| Diabetes, n (%) | 80 (40.6) | 35 (53.0) | 39 (52.0) | 113 (54.9) | 0.028 |
| Obstructive CAD, n (%) | 98 (49.7) | 34 (51.5) | 40 (53.3) | 100 (48.5) | 0.902 |
| Smoking status, n (%) | 61 (31.0) | 13 (19.7) | 33 (44.0) | 64 (31.1) | 0.021 |
| SBP, mmHg | 142.7 ± 18.3 | 144.0 ± 12.0 | 137.6 ± 14.6 | 142.7 ± 17.8 | 0.084 |
| DBP, mmHg | 77.4 ± 10.3 | 77.5 ± 9.1 | 77.4 ± 9.4 | 79.7 ± 10.2 | 0.091 |
| WBC count, ×10E9/L | 6.97 ± 1.61 | 6.78 ± 1.89 | 6.91 ± 1.32 | 6.99 ± 1.72 | 0.817 |
| Creatinine, μmol/L | 80.15 ± 20.51 | 71.87 ± 21.29 | 80.64 ± 20.50 | 78.06 ± 20.38 | 0.030 |
| eGFR, mL/min/1.73m^2^ | 79.56 ± 16.71 | 85.29 ± 16.56 | 82.33 ± 18.18 | 83.53 ± 18.26 | 0.053 |
| Uric acid, mmol/L | 364.81 ± 115.31 | 342.23 ± 101.07 | 386.33 ± 110.75 | 395.52 ± 103.63 | 0.001 |
| FPG, mmol/L | 6.19 ± 4.17 | 6.46 ± 2.50 | 6.80 ± 2.87 | 6.85 ± 3.20 | 0.245 |
| HbA1c, (%) | 6.50 ± 1.58 | 6.88 ± 1.69 | 6.87 ± 1.64 | 6.96 ± 1.87 | 0.055 |
| TC, mmol/L | 3.94 ± 0.64 | 5.65 ± 0.84 | 4.28 ± 0.54 | 5.86 ± 0.06 | < 0.001 |
| TG, mmol/L | 1.11 (0.85, 1.49) | 1.78 (1.21, 2.46) | 1.58 (1.09, 2.37) | 1.94 (1.41, 2.83) | < 0.001 |
| HDL-C, mmol/L | 1.15 ± 0.30 | 1.22 ± 0.35 | 1.04 ± 0.28 | 1.11 ± 0.26 | 0.001 |
| LDL-C, mmol/L | 2.37 ± 0.57 | 3.47 ± 0.69 | 2.60 ± 0.52 | 3.81 ± 0.87 | < 0.001 |
| Lipoprotein(a), mg/L | 131.80 (72.45, 242.50) | 135.10 (79.98, 247.38) | 128.60 (60.00, 201.90) | 128.60 (69.00, 271.75) | 0.708 |
| Non-HDL-C, mmol/L | 2.79 ± 0.54 | 4.43 ± 0.80 | 3.24 ± 0.44 | 4.75 ± 0.81 | < 0.001 |
| ApoB, g/L | 0.75 ± 0.14 | 0.85 ± 0.11 | 1.19 ± 0.15 | 1.39 ± 0.39 | < 0.001 |
| Anti-platelet medication, n (%) | 175 (88.8) | 58 (87.9) | 60 (80.0) | 180 (87.4) | 0.273 |
| Statins, n (%) | 175 (88.8) | 52 (78.8) | 59 (78.7) | 180 (87.4) | 0.054 |
| β-blocker, n (%) | 84 (42.6) | 28 (42.4) | 22 (29.3) | 84 (40.8) | 0.227 |
| ACEI/ARB, n (%) | 121 (61.4) | 41 (62.1) | 42 (56.0) | 113 (54.9) | 0.502 |
| Glucose-lowering therapy, n (%) | 69 (35.0) | 29 (43.9) | 34 (45.3) | 88 (42.7) | 0.270 |
| Initial GS | 7.0 (3.8, 13.0) | 5.0 (2.9, 12.3) | 6.0 (4.5, 15.0) | 7.0 (3.9, 14.0) | 0.774 |

Data are mean ± SD, or median with interquartile range, or frequencies with percentage, as appropriate.

*ApoB* apolipoprotein B, *BMI* body mass index, *CAD* coronary artery disease, *SBP* systolic blood pressure, *DBP* diastolic blood pressure, *WBC* white cell counts, *eGFR* estimated glomerular filtration rate, *FPG* fasting plasma glucose, *HbA1c* hemoglobinA1c, *TC* total cholesterol, *TG* triglycerides, *HDL-C* high-density lipoprotein cholesterol, *LDL-C* low-density lipoprotein cholesterol, *ACEI* angiotensin converting enzyme inhibitor, *ARB* angiotensin receptor blocker, *GS* Gensini Score.
