# Supplementary material for: Apolipoprotein B is associated with CT-angiographic progression beyond low-density lipoprotein cholesterol and non-high-density lipoprotein cholesterol in patients with coronary artery disease
Source: Lipids Health Dis. 2023 Aug 9;22:125. doi: 10.1186/s12944-023-01872-6 (PMC10410799; doi:10.1186/s12944-023-01872-6)
Supplement: Supplementary file 3 — Supplementary Material 3 [file 12944_2023_1872_MOESM3_ESM.pdf]

PAPER NAME

Apolipoprotein B is associated with CT-angiographic progression beyond low-density lipoprotein chole

WORD COUNT	CHARACTER COUNT
3495 Words	20332 Characters
PAGE COUNT	FILE SIZE
16 Pages	38.0KB
SUBMISSION DATE	REPORT DATE
Jun 4, 2023 9:52 PM GMT+8	Jun 4, 2023 9:53 PM GMT+8

16% Overall Similarity

The combined total of all matches, including overlapping sources, for each database.

- 11% Internet database
- 15% Publications database
- Crossref database
- Crossref Posted Content database
- 0% Submitted Works database

Apolipoprotein B is associated with CT-angiographic progression beyond low-density lipoprotein cholesterol and non-high-density lipoprotein cholesterol in patients with coronary artery disease

Abstract

Background: Accumulating evidence indicated that apolipoprotein B (apoB) was the principal lipid determinant of coronary artery disease (CAD). Nevertheless, the connection between apoB and angiographic progression of CAD remained undetermined.

Methods: Five hundred and forty-four CAD patients with twice coronary computed tomography angiography experiences were enrolled. The Gensini scoring system was used to assess angiographic progression. Incident angiographic progression was defined as an annual change rate of the Gensini score of > 1 point. The predictive efficacy of baseline apoB levels for angiographic progression was assessed using a receiver operating characteristic (ROC) curve. For comparative purposes, patients were categorized into three sets according to their baseline apoB tertiles. Furthermore, discordance analyses defined by the median were performed to assess the superiority of apoB over lipoprotein cholesterol in predicting angiographic progression of CAD.

Results: Angiographic progression was observed in 184 patients (33.8%) during a follow-up period of 2.2-year. The area under the ROC curve was 0.565 (0.522 - 0.607, *P* = 0.013). The incidence of angiographic progression was elevated with increasing apoB tertile after adjusting for confounding factors [odds ratio (OR) for the medium

apoB tertile: 1.92, 95% confidence interval (CI): 1.15 - 3.19, *P* = 0.012; OR for the high apoB tertile: 2.05, 95%CI: 1.17 - 3.60, *P* = 0.013]. Additionally, discordance analyses showed that the higher apoB group had a significantly higher risk of CAD progression in the fully adjusted model (all $P < 0.05$).

Conclusions: ApoB could be used as an accurate and comprehensive indicator of angiographic progression in patients with CAD.

Keywords: apolipoprotein B, low-density lipoprotein cholesterol, non-high-density lipoprotein cholesterol, coronary artery disease, angiographic progression

Background

Although lowering of plasma low-density lipoprotein cholesterol (LDL-C) has been recognized to substantially decrease the risk of coronary artery disease (CAD), recent studies have shown a high residual cardiovascular risk after single LDL-C intervention [1-3]. Because the deposition and accumulation of LDL-C within the arterial lumen were the principal determinants of atherosclerosis, lowering LDL-C was strongly recommended by current guidelines [4, 5]. Though achieved guideline-recommended LDL-C goals with lipid lowering treatment, 22.7% of the enrolled patients in the PROVE-IT 22 trial continued to experience major cardiovascular-related events during the two-year follow-up [6]. Thus, a focus solely on LDL-C levels was insufficient as an optimal strategy for risk evaluation and management of CAD.

Apolipoprotein B (apoB) was the critical structural protein in the low-density lipoprotein (LDL) and triglyceride (TG)-rich lipoproteins [7]. Recently, Ference et al. demonstrated that lowering the amount of very low-density lipoprotein (VLDL) and LDL particles yielded a similar decrease in cardiovascular risk, indicating that both were equally atherogenic [8]. Thus, compared with the variable cholesterol content within them, the quantity of apoB-containing lipoprotein particles was considered a more comprehensive indicator of atherogenic risk [9]. Indeed, evidence from Mendelian randomization (MR) and discordance analyses revealed that apoB was a more precise biomarker in risk estimation and medication guidance compared with LDL-C and non-high-density lipoprotein cholesterol (non-HDL-C) alone [10, 11]. The effectiveness of apoB propelled its recommendation for risk assessment of CAD in ESC/EAS 2019

guidelines [5]. Notwithstanding its significance, the large data gap between apoB and CAD progression impeded its application. Furthermore, the advantage of apoB over usual biomarkers in estimating the angiographic progression (AP) of CAD remained debatable.

Hence, the current study aimed to elucidate the relation of apoB with AP in individuals diagnosed with CAD using coronary computed tomography angiography (CTA). A discordance analysis was performed to evaluate the effectiveness of apoB against other biomarkers in forecasting the possibility of AP.

Methods

Patients

Patients who underwent a series of non-invasive coronary angiography assessments of coronary CTA between January 2009 and December 2015 at the Third Affiliated Hospital, Sun Yet-sen University, were retrospectively reviewed. Initially, 1242 patients received coronary CTA twice with an interval of ⩾ 6 months because of angina-like symptoms, abnormal ST-T segment changes on electrocardiography, abnormal regional wall motion on echocardiography, and routine monitoring to seek anatomical progression. Of these, patients with no CAD (recognized as having no prior reported CAD and no luminal stenosis on initial coronary CTA) were excluded [12]. Patients who had undergone coronary revascularization with interventional therapy or surgery were considered ineligible. Patients with malignancy or severe dysfunction of liver and kidney [estimated glomerular filtration rate (eGFR) < 30 mL/minutes/1.73m^2, or those

with hemodialysis or peritoneal dialysis] were also eliminated from the study. Finally, 544 patients were analyzed (as shown in Fig. 1). Among them, 72 (13.2%) patients received repeated CTA in 1-year because of new or recurrent ischemic symptoms, while the others underwent repeated CTA for routine follow-up during the 2.5-year (interquartile range: 1.7 - 3.6).

Anthropometric and Risk Factor Measurements

Information on demographics, height, weight, blood pressure (BP), smoking habits, case history and drug usage was collected during the initial coronary CTA examination. Body mass index (BMI) was obtained from weight/height squared (kg/m^2). Hypertension (HTN) definition was adopted from a previous study [13]. Diagnostic criteria for diabetes mellitus (DM) were also adopted from a previous study [14]. Participants who smoked ⩾ 1 cigarette per day within 1-year prior to the present study were considered current smokers.

Clinical Chemistry Parameters

Venous blood was collected after fasting for 12 h and measured in the laboratory. Baseline lipid profiles, FPG, uric acid (UA), liver function, and renal function were assessed using a biochemical analyzer (Hitachi 7600, Tokyo, Japan). Direct methods were used to detect LDL-C concentrations, as well as HDL-C. Total cholesterol (TC) as well as TG levels were analyzed by enzyme colorimetry. Lipoprotein(a) levels were assessed using latex immunoturbidimetry. ApoB concentrations were detected using

immunoturbidimetry (assay kit, cat. No CH0101158, Tokyo, Japan). Non-HDL-C was defined as TC level minus HDL-C level.

Non-invasive Coronary Angiography Assessments and Scoring

Coronary CTA was performed using a 320-row multi-detector computed tomography (TOSHIBA, Tokyo, Japan) based on the recommendations in previous study [15]. The standard operating procedure was pre-fixed as previously described [16]. All images were reconstructed by an experienced cardiac radiologist (workstation: VitreafX Version 2.1, TOSHIBA, Tokyo, Japan) according to the Society of Cardiovascular CT (SCCT) guidelines [15]. CAD was diagnosed by initial coronary CTA examination and graded as no CAD (recognized as having no prior reported CAD and no luminal stenosis), non-obstructive CAD (defined as 1% - 49% of luminal diameter stenosis in an epicardial coronary artery), or obstructive CAD (considered as ≥ 50% stenosis) by visual evaluation [12].

The lesion of the coronary artery was scored using the Gensini score (GS) and considering the importance of the coronary artery, location of stenosis, and extent of luminal stenosis [17]. The calculation was performed by two cardiologists, and inconsistencies were determined by a third cardiologist. Incident AP was defined as an annual change rate of GS > 1 point, as described in a previous study [18]. Otherwise, those with the annual change rate of GS ≤ 1 point were allocated into the non-progression group.

Statistical Analysis

SPSS 23.0 software for Windows (IBM Corp., Armonk, NY, USA) was applied for data analyses. Normal and skewed distributed data were displayed as mean ± standard deviation (SD) and interquartile range, respectively. Categorical variables were showed as frequencies (percentages). Between-group differences were carried out by X^2 test, analysis of variance, or Kruskal-wallis rank test. ApoB performance in predicting progression was evaluated using the receiver operating characteristic (ROC) curve in Medcalc 20.0.3 software (MedCalc, Ostend, Belgium). For logistic regression analysis, patients were assigned to the low, the medium, and the high group according to baseline apoB and LDL-C tertiles. Two logistic regression models with different levels of adjustment were established for the relationship between incident AP and baseline levels of apoB and LDL-C, using the low apoB and LDL-C group as reference, respectively. Significant variables ($P < 0.1$) in the univariate logistic regression analysis were selected for the models. Model 1 was adjusted for baseline age, BMI, sex, smoking status, HTN, DM, and obstructive CAD. Model 2 was additionally adjusted for hemoglobin A1c (HbA1c), HDL-C, lipoprotein(a), initial GS, statins and β blocker used at baseline. Owing to its clinical importance, baseline LDL-C level was additionally included when adjusting for confounding factors in Model 2 to assess the performance of baseline apoB in predicting AP.

To further evaluate whether baseline apoB levels were more effective than baseline LDL-C and non-HDL-C levels in predicting AP, discordance analyses were performed as described in previous study [19]. Median cutoff points were chosen to define

discordance: apoB below (<) the median and LDL-C at or above (≥) the median, or vice versa. In brief, participants were categorized into four groups according to their median values: low apoB/low LDL-C, low apoB/high LDL-C, high apoB/low LDL-C, and high apoB/high LDL-C. Similarly, four concordant/discordant categorizes of apoB and non-HDL-C were created. The characteristics of these categories were analyzed. Using the same fully adjusted model (Model2), the association between the concordant/discordant groups of apoB and LDL-C or non-HDL-C and incident AP was evaluated, using the low/low group as a reference. $P < 0.05$ was defined statistically significant.

Results

The average age of the patients at baseline was 64.4 years: 56.4% were male and 43.6% were females. Overall, 72.6% had HTN, 49.1% had DM, 50.0% had obstructive CAD, 31.4% were current smokers, and 85.7% had received statins therapy at baseline. The demographic and clinical comorbidities, metabolic parameters, and medications of patients according to tertiles of baseline apoB were showed in Table 1. Patients who underwent repeated CTA within 1-year were comparable among the groups (14.4%, 13.2%, and 12.1% for the low, the medium, and the high apoB group, respectively; $P = 0.803$). Patients with high apoB levels were younger, and had more severe metabolic abnormalities, including higher baseline BMI, TC, non-HDL-C, UA, FPG, HbA1c, TG, and LDL-C, than those with low or medium apoB levels. However, HDL-C concentrations declined with elevated apoB tertiles.

Discordance was defined according to the median values. The median values at baseline were 1.00 g/L for apoB, 3.02 mmol/L for LDL-C, and 3.76mmol/L for non-HDL-C. Relative to LDL-C, the prevalence of low apoB/high LDL-C and high apoB/low LDL-C levels were 15.1% and 16.7%, respectively. Age, BMI, creatinine, eGFR, UA, TC, TG, HDL-C, non-HDL-C, and lipoprotein(a) levels were significantly different between the four concordance/discordance groups, as listed in Table 2. Relative to non-HDL-C, the proportions of patients with low apoB/ high non-HDL-C and high apoB/low non-HDL-C levels were 12.1% and 13.8%, respectively. Significant differences attributed to age, BMI, prevalence of DM, smoking status, UA levels, and lipid profiles were observed among the four groups and was listed in Supplementary Table 1.

ROC curve was conducted to identify the efficacy of baseline apoB levels in predicting AP. The area under the ROC curve was 0.565 (0.522 - 0.607, $P = 0.013$). (Fig.2)

During the 2.2-year (interquartile range: 1.4 - 3.4) of follow-up, AP was found in 184 patients (33.8%). The relations of baseline apoB as well as LDL-C tertiles with AP were analyzed using multivariate logistic regression (Table 3). Compared with the low level of apoB group (reference), the odds ratio (OR) [95% confidence interval (CI)] of AP for the medium and high level of apoB was 1.42 (0.90 - 2.22) and 1.75 (1.12 - 2.72), respectively. The adjusted OR (95% CI) of the medium and high level of apoB was 1.83 (1.14 - 2.96) and 2.23 (1.38 - 3.61), respectively, for the risk of AP (all $P < 0.05$) in Model 1. After additionally adjusting for HbA1c, HDL-C, lipoprotein(a), initial GS, as

well as statins and β blocker used at baseline in Model 2, the associations remained statistically significant (OR for the medium tertile: 1.93, 95%CI: 1.18 - 3.16, $P = 0.009$; OR for the high tertile:2.08, 95%CI: 1.27 - 3.43, $P = 0.004$). However, the results remained the same even after adjustment of baseline LDL-C in Model 2 (OR for the medium tertile: 1.92, 95%CI: 1.15 - 3.19, and $P = 0.012$; OR for the high tertile:2.05, 95%CI: 1.17 - 3.60, and $P = 0.013$). When stratified by baseline LDL-C tertile, elevated LDL-C levels showed no correlation with the risk of progression.

Using the low apoB/low LDL-C group as a reference in the logistic regression model, discordance analysis was performed to evaluate the effectiveness of baseline apoB and LDL-C. Results were listed in Table 3. Compared with the reference in the model with full adjustments (Model 2), the adjusted OR for AP of high apoB/low LDL-C group and high apoB/high LDL-C group was 2.05 (95% CI:1.11 - 3.78, $P = 0.022$) and 2.32 (95% CI:1.42 - 3.81, $P = 0.001$), respectively. Contrastingly, in the low apoB/high LDL-C group, association to AP was not observed (OR:1.72, 95% CI: 0.94 - 3.16, $P = 0.079$). Similar relationships were explored across the concordance and discordance between apoB and non-HDL-C levels. Patients in the high apoB/low non-HDL-C and high apoB/high non-HDL-C groups were significantly correlated with AP (all $P < 0.05$), as opposed to patients in the low apoB/high non-HDL-C group, who had no correlation with AP. Results were displayed in Fig.3.

Discussion

This study suggested that incremental apoB levels were positively correlated with the

incidence of AP in CAD patients apart from traditional risk factors and statins treatment. In the model with full adjustments, discordance analyses showed a significantly higher risk of AP in the higher apoB groups regardless of non-HDL-C or LDL-C levels. Thus, apoB was a more accurate and comprehensive indicator of AP than non-HDL-C and LDL-C in CAD patients.

Previously, LDL-C and VLDL-C were identified as biomarkers for cardiovascular risk and treatment targets; however, recent studies revealed their insufficiency [20, 21]. Many individuals with normal or guideline-recommended LDL-C levels experienced cardiovascular events or progression of atherosclerosis [22]. Recent MR studies determined that the atherogenic risk from a VLDL particle was similar to that from an LDL particle. Because of dynamic lipid metabolic processes and the inability of plasma LDL-C and TG levels to reflect the actual number of LDL and VLDL particles [8, 23], neither LDL-C nor TG were perfect indicators of cardiovascular risk.

Recently, mounting evidence showed that the quantity of apoB particles that entered and were retained within the arterial wall was the principal determinant of atherosclerosis [7,9]. Though cholesterol deposition within the arterial lumen was regarded as a classic characteristic of atherosclerosis, it could not enter the arterial wall unless it was carried by apoB particles [7]. Smaller cholesterol-poor apoB particles could be easily retained within the arterial wall than larger cholesterol-rich ones because of their high affinity for glycosaminoglycans, whereas more cholesterol was deposited when larger cholesterol-rich apoB particles were retained. The net result indicated that each apoB particle was proatherogenic [24]. Furthermore, phospholipids, one of the

components of apoB particles, were strong proatherogenic factors and associated with poor prognosis in an oxidized state [25]. Thus, compared to LDL-C, apoB particles integrated more proatherogenic risk factors in most cases. Because each proatherogenic apoB particle contained only one molecule of apoB, apoB levels could provide a measure of the total levels of proatherogenic lipoprotein particles [26].

Growing evidence illustrated the importance of apoB in the prevalence of CAD [25, 27]; however, its predictive efficacy for CAD progression remained unclear. Richardson et al. performed a genome-wide association study of LDL-C, apoB, and TG in the UK Biobank and validated the associations using a second database, CARDIoGRAMplusC4D. When evaluated individually using MR, LDL-C, apoB and TG levels were positively correlated with the incidence of CAD. Nevertheless, only apoB retained a robust positive association with multivariable MR [10]. Similarly, Zuber et al. confirmed apoB as the principal lipid detriment of CAD in multivariable MR models [28]. However, few real-world evidence studies assessed the relationship between apoB levels and progression of coronary atherosclerosis. Ohwada et al. identified that apoB levels had association with the necrotic core volumes in culprit coronary artery lesions and plaque advancement in 115 patients with stable CAD [29]. Subsequently, Kim et al. found a dose-response relationship between baseline apoB levels and coronary artery calcification progression [30]. To further explore the effectiveness of apoB in secondary prevention, a retrospective study including 544 patients with CAD was conducted using a coronary CTA. 85.7% of them initially received statins therapy. In this present study, it was observed that the incidence of AP

increased with an increasing number of apoB tertiles during follow-up.

However, there was no association between the progression risk and elevated LDL-C levels while receiving statins therapy, which was in line with a previous study conducted by Yao et al. [31]. They concluded that higher apoB levels, rather than LDL-C levels were significantly correlated with new-onset CAD in participants who received statins therapy. Furthermore, Ference et al. suggested that the absolute reduction in the amount of apoB-containing lipoprotein particles, rather than the expected per-unit change in LDL-C, was a superior marker for predicting the cardiovascular events risk [32]. Moreover, Baik. et al. showed that apoB was positively correlated with symptomatic intracranial atherosclerotic stenosis, regardless of pre-admission statins use, whereas LDL-C was not, indicating apoB may be superior to LDL-C for residual risk evaluation under statins therapy [33]. Generally, LDL-C and apoB levels had closely correlation as well as similar information about atherogenic risk. When they became discordant, the predictive values of atherogenic risk differentiated. Under statins treatment, the LDL-C concentration may underestimate the residual atherogenic risk. However, apoB levels could integrate more atherogenic risk factors (including VLDL and its remnants) and serve as a comprehensive biomarker for risk evaluation. The present study identified that apoB levels could be used to monitor the progression of CAD in secondary prevention, even under statins treatment.

In this study, median values defined discordance analyses demonstrated the advantage of apoB in predicting AP progression. Regardless of LDL-C/non-HDL-C levels, elevated apoB levels were associated with AP risk in patients with CAD. This

was consistent with results obtained by Johannesen et al., who also employed medians to define the discordance analysis. They found that elevated apoB levels were correlated with all-cause mortality regardless of LDL-C and non-HDL-C levels [34]. Lawler et al. conducted a discordance analysis using 27,533 apparently healthy women to identify whether the count of apoB particles or the cholesterol amount could predict incident CAD risk [35]. Compared with non-HDL-C, they found that apoB levels had a stronger relationship with CAD risk. Overall, apoB could be a potential biomarker for cardiovascular risk prediction for both primary and secondary preventions. Application of lipidomic technologies could provide deeper insight into the correlation between CAD progression and the lipid composition of atherogenic particles [36]. Therefore, further studies are required to validate these findings using lipidomic approach to identify more precise components for monitoring and guiding the individualized therapy.

ApoB was considered not only an atherogenic biomarker, but also a therapeutic target. Recent literatures supported the hypothesis that reducing apoB levels led to a decrease in risk of cardiovascular diseases [9]. The higher the concentration of autoantibodies against apoB in the plasma, the lower the rate of coronary atherosclerosis-related diseases [37]. Khan, et al. found that an absolute decrease in apoB was correlated with a decrease in cardiovascular events in a meta-analysis study [38]. A more recent study by Hagström, et al. demonstrated that in patients with recent acute coronary syndrome, a higher baseline apoB level was associated with a higher incidence of major adverse cardiovascular events (MACE). Under alirocumab treatment, lower apoB levels

resulted in a lower risk of MACE, even with guideline-recommended LDL-C or non-HDL-C levels [39]. Thus, lowering apoB levels with targeted therapy was a potential therapeutic strategy for CAD and further study employing small compounds or monoclonal antibodies screening are warranted.

Study strengths and Limitations

This retrospective longitudinal study included 544 patients with a relatively short follow-up period. With strict statistical adjustment, this real-world evidence study revealed that apoB was an effective biomarker for predicting disease progression in secondary prevention of CAD. The finding provided additional evidence to support the role and effectiveness of apoB in identifying lipid atherogenic risks and guiding individualized therapy in clinical practice.

Nonetheless, the study had some limitations. First, potential residual confounding factors were difficult to exclude because this was a retrospective, observational study design. Second, although the use of statins at baseline were adjusted for, information on adherence and changes in intensity dosage during follow-up were not confirmed. Third, the present study was a small sample size study with a relatively short follow-up period.

Conclusions

A positive association between high apoB levels and the incidence of AP in patients with CAD was observed after adjusting for LDL-C or non-HDL-C levels. Therefore,

apoB could be used as an accurate and effective biomarker for risk evaluation of patients with CAD in secondary prevention. This study added to the literature on the role and effectiveness of apoB in identifying the lipid atherogenic risks. The importance of apoB particles in the initiation and progression of CAD will gradually be appreciated, and routine measurement of apoB should be advised to guide risk stratification and therapy in clinical practice.

16% Overall Similarity

Top sources found in the following databases:

- 11% Internet database
- 15% Publications database
- Crossref database
- Crossref Posted Content database
- 0% Submitted Works database

TOP SOURCES

The sources with the highest number of matches within the submission. Overlapping sources will not be displayed.

#	Source	Database	Similarity
1	jstage.jst.go.jp	Internet	2%
2	Xing Shui, Zefeng Chen, Zheqi Wen, Leile Tang, Wenyu Tang, Yixuan Li...	Crossref	1%
3	hdl.handle.net	Internet	<1%
4	"ESICM LIVES 2017", Intensive Care Medicine Experimental, 2017	Crossref	<1%
5	Wilkins, John T., Ron C. Li, Allan Sniderman, Cheeling Chan, and Donal...	Crossref	<1%
6	Sniderman, Allan D., Peter P. Toth, George Thanassoulis, and Curt D. F...	Crossref	<1%
7	academic.oup.com	Internet	<1%
8	"Therapeutic Lipidology", Springer Science and Business Media LLC, ...	Crossref	<1%

9	researchonline.ljmu.ac.uk<br>Internet	<1%
10	ncbi.nlm.nih.gov<br>Internet	<1%
11	link.springer.com<br>Internet	<1%
12	Tamara Glavinovic, Allan D. Sniderman. "Apolipoprotein B: the Rosetta ...<br>Crossref	<1%
13	researchsquare.com<br>Internet	<1%
14	science.gov<br>Internet	<1%
15	Lasse Jespersen, Steen Z. Abildstrøm, Anders Hvelplund, Eva Prescott...<br>Crossref	<1%
16	jeccr.biomedcentral.com<br>Internet	<1%
17	nice.org.uk<br>Internet	<1%
18	Ballantyne, C.M.. "Comparison of the efficacy and safety of a combinat...<br>Crossref	<1%
19	Geyue Qu, Zhongying Zhang, Hong Zhu. "Discordance between apolipo...<br>Crossref	<1%
20	pure.amc.nl<br>Internet	<1%

21	cancerjournal.net<br>Internet	<1%
22	Yuriko Abe, Tomoo Okada, Reiko Sugiura, Kuniaki Yamauchi, Mitsunori...<br>Crossref	<1%
23	clinicaltrials.gov<br>Internet	<1%
24	nature.com<br>Internet	<1%
25	Brian A. Ference. "Causal Effect of Lipids and Lipoproteins on Atheros...<br>Crossref	<1%
26	bmcophthalmol.biomedcentral.com<br>Internet	<1%
27	researchgate.net<br>Internet	<1%
28	Gouri, A., A. Dekaken, A. Yakhlef, A.A. Bentorki, and N. Kouicem. "Non-...<br>Crossref	<1%
29	journals.sagepub.com<br>Internet	<1%
30	scholarworks.waldenu.edu<br>Internet	<1%
31	worldwidescience.org<br>Internet	<1%
32	omicsdi.org<br>Internet	<1%

33	"Therapeutic Lipidology", Springer Science and Business Media LLC, ...<br>Crossref	<1%
34	Chan-Won Kim, Sungwoo Hong, Yoosoo Chang, Jung Ah Lee, Hocheol ...<br>Crossref	<1%
35	G. WALLDIUS. "The apoB/apoA-I ratio: a strong, new risk factor for car...<br>Crossref	<1%
36	Okada, Katsuki, Yasunori Ueda, Tadateru Takayama, Junko Honye, Sei ...<br>Crossref	<1%
37	"Cardiovascular Risk Assessment in Primary Prevention", Springer Scie...<br>Crossref	<1%
38	Chunyan Zhang, Jingwei Ni, Zhenyue Chen. "Apolipoprotein B Displays ...<br>Crossref	<1%
39	Fitzgerald, Richard Joaquim. "Optimisation of Anti-Platelet Therapy in ...<br>Publication	<1%
40	Francis, Michael. "Nutrigenetics of Polyunsaturated Fatty Acids and Ve...<br>Publication	<1%
41	Richard J. Webb, Mohsen Mazidi, Gregory Y.H. Lip, Andre P. Kengne, M...<br>Crossref	<1%
